# Mental Health Status of Paediatric Medical Workers in China During the COVID-19 Outbreak

**DOI:** 10.3389/fpsyt.2020.00702

**Published:** 2020-07-21

**Authors:** Yin Liu, Li Wang, Long Chen, Xianhong Zhang, Lei Bao, Yuan Shi

**Affiliations:** ^1^ Department of Neonatology, Ministry of Education Key Laboratory of Child Development and Disorders, National Clinical Research Center for Child Health and Disorders, China International Science and Technology Cooperation Base of Child Development and Critical Disorders, Children’s Hospital of Chongqing Medical University, Chongqing Key Laboratory of Pediatrics, Chongqing, China; ^2^ Department of Pediatrics, Daping Hospital, Army Medical University, Chongqing, China

**Keywords:** mental health status, paediatric medical workers, COVID-19, outbreak, China

## Abstract

**Background:**

A novel coronavirus (COVID-19) outbreak has occurred in China, and national medical workers have been thrown into this silent battle. Paediatric medical workers have been an important part of this battle and under enormous pressure. This paper evaluates the depression, anxiety, and stress of paediatric medical staff during the epidemic and examines related impact factors.

**Methods:**

We conducted this study using online questionnaires *via* social networking software during the week of Feb. 17 to Feb. 23, 2020. The 21-item Depression Anxiety Stress Scale (DASS), which is a revised, simplified version of the original DASS developed by Lovibond et al., was used in this study.

**Results:**

Among all 2,031 respondents, 14.81%, 18.3%, and 9.98% had depression, anxiety and stress symptoms, respectively. Males, doctors, individuals aged between 31‑60 years, those with senior job titles, those who had contact with patients with confirmed or suspected cases of COVID-19, those who worked on the clinical frontlines fighting the epidemic and those who had experience combating similar outbreaks were more likely to have depression, anxiety or stress symptoms. Respondents in Beijing and Chongqing had lower negative psychological symptom scores than the national average.

**Conclusion:**

During the COVID-19 outbreak, depression, anxiety and stress are present to varying degrees among paediatric medical workers across the country. Psychological crisis interventions should be implemented to protect the mental health of paediatric medical workers during and after the epidemic.

## Introduction

In December 2019, a novel coronavirus outbreak occurred in Wuhan, Hubei, China. The virus has rapidly spread across China and to at least 212 countries (1). By March 6, 2020, 80,585 confirmed cases (nearly 500 child cases) and 522 suspected cases, among which 52,297 cases were cured and 3,016 were fatal, were reported in all provinces of China. Since the outbreak, China’s health authorities have taken a series of actions to control the spread of infectious diseases. However, during this dangerous epidemic situations, medical treatment resources were in short supply. Medical staff were exhausted, and facing enormous pressure (2).

Previous studies have shown that during and after the outbreak of SARS in 2003, medical workers suffered from different degrees of psychological distress, such as stress, anxiety, and depressive symptoms, due to a lack of mental health care. These mental health problems not only affect medical workers’ attention, understanding, and decision-making ability, which might hinder the fight against COVID-19, but also could have a lasting effect on their overall wellbeing. Therefore, psychological crisis intervention is another important task in the fight against COVID-19. The Chinese government has incorporated psychological crisis intervention into the overall efforts for epidemic prevention and control (3), but because of the relatively low incidence of COVID-19 infection in children, psychological intervention for paediatric medical staff has not attracted the attention of the government and relevant departments. Thus far, there is no relevant report on protective measures for the mental health of paediatric medical workers.

According to the “White Paper on the Current Situation of Paediatric Resources in China” issued by the paediatric branch of the Chinese Medical Association in 2017, the number of paediatric medical staff in China is seriously insufficient. This deficit is associated with high occupational risk, conflict between doctors and patients, heavy workload, long working hours, and low pay, among other factors. Because of this medical environment, paediatric medical staff experience greater long-term psychological pressure than other Chinese medical workers. In addition, respiratory tract infection is a common clinical disease in children, and more than half of paediatric outpatients have fever and cough symptoms, which are also common symptoms of COVID-19. After the outbreak of the epidemic, due to fear of the disease, a large number of children with fever and cough symptoms poured into paediatric clinics, making paediatric medical resources more scarce and placing paediatric medical staff under great pressure. Protecting the mental health of paediatric medical workers during and after the epidemic is thus important for the control of the epidemic and for workers’ own long-term health. An investigation of the mental health status of paediatric medical staff is the first step.

In this study, 2031 paediatric medical workers in China were surveyed to understand the severity of depression, anxiety, and stress among such workers during the epidemic in order to provide a scientific basis for psychological protection and intervention.

## Method

### Participants

To collect data, this study used a web-based cross-sectional survey based on the Internet Survey of Chinese Medical Doctor Association (CMDA), an ongoing, online mental health-related survey of Chinese paediatric medical workers. This web-based survey was sent to several WeChat working groups of paediatric medical staff involved in the CMDA. Paediatric medical workers answered the questionnaire by scanning the Quick Response code (QR code) of the questionnaire address or clicking a link. Two members of the research team answered participants’ questions online during the survey completion process. To encourage participation, all participants could receive reports about their mental health after completing the survey. This survey was completely voluntary and non-commercial. The doctors and nurses who were interviewed were told that the results of the survey would be used for scientific research, but they were not informed of the specific research plan. All respondents were anonymous.

### Questionnaire Measures and Procedure

**Figure 1:**
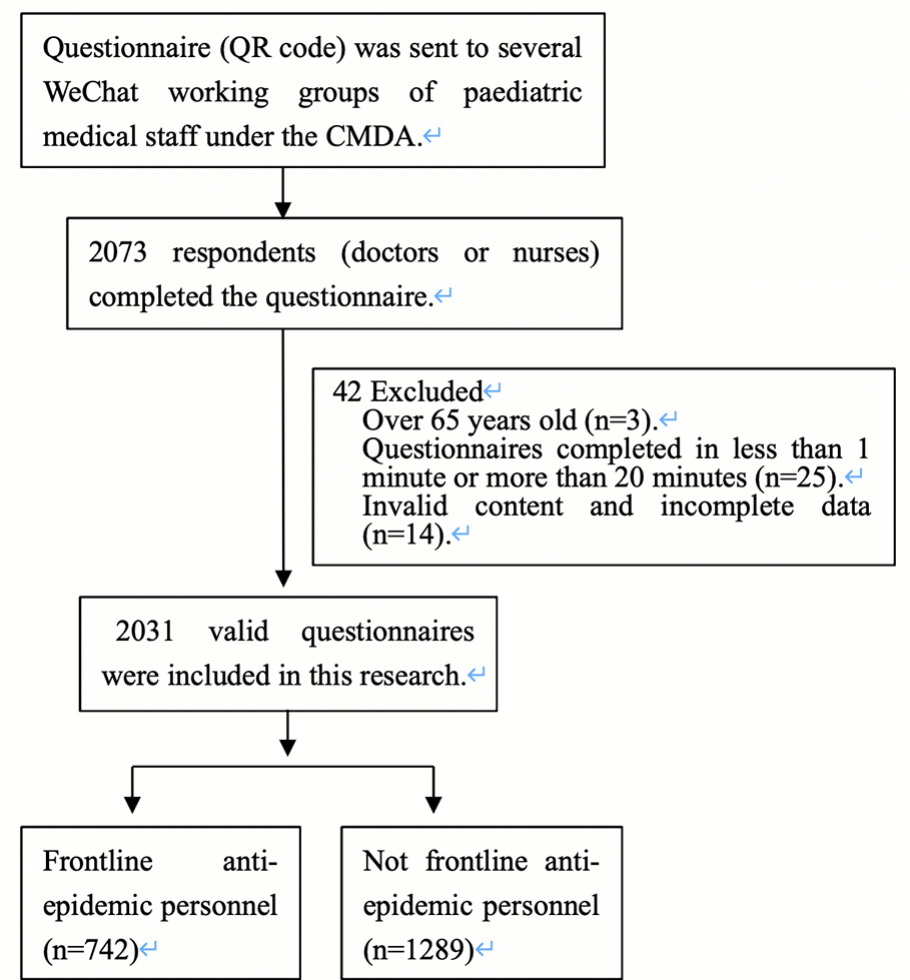
Flow diagram of selection of the participants.

Participants answered the questionnaires anonymously on the Internet from Feb. 17 to Feb. 23, 2020. The questionnaire included questions on sociodemographic characteristics, anxiety symptomology, depressive symptomology, and stress symptomology. Sociodemographic characteristics included gender, age, identity (doctor/nurse), professional title, education, health condition, area of work during the coronavirus outbreak, experience with similar public health events and frontline anti-epidemic personnel status (exposure to patients with confirmed or suspected cases of COVID-19). A total of 2,073 respondents (doctors or nurses) completed the questionnaire. To ensure the quality of the survey, we set the following exclusion criteria: over 65 years old, questionnaires completed in less than 1 min or more than 20 min, invalid content and incomplete data. Finally, a total of 2,031 valid questionnaires were included in this research (**Figure 1** shows the case selection algorithm).

The 21-item Depression Anxiety Stress Scale (DASS), which is a revised, simplified version of the original DASS developed by Lovibond et al., was used in this study (4). The DASS-21 has the same reliability and validity as the full version (5). The Chinese version of the DASS-21 was used in this study (6, 7). The DASS-21 includes three subscales with a total of 21 items that investigate the degree of depression, anxiety and stress. Items are scored on a 4-point scale ranging from 0‑3, where 0 is never, 1 is sometimes, 2 is often, and 3 is almost always or always. The sum of the item scores for each subscale multiplied by 2 is the subscale score, which ranges from 0–42 points. For the Depression subscale, a score of ≤ 9 points is normal, while a score of 10-l3 points indicates mild depression, 14–20 points moderate depression, 21–27 points severe depression, and ≥ 28 points very serious depression. For the Anxiety subscale, a score of ≤ 7 points is normal, while 8‑9 points indicates mild anxiety, 10–14 points moderate anxiety, 15–l9 points severe anxiety, and ≥ 20 points very serious anxiety. For the Stress subscale, a score of ≤ 14 points is normal, while 15–l8 points indicates mild stress, 19–25 points moderate stress, 26–33 points severe stress, and ≥ 34 points very serious stress. The higher the score is, the more serious the degree of depression, anxiety or stress (5). Cronbach’s alpha coefficient of this questionnaire is 0.912.

### Ethical Statement

This study was approved by the Ethics Committee of Children’s Hospital of Chongqing Medical University. Electronic informed consent was obtained from each participant prior to starting the investigation. Participants could withdraw from the survey at any moment without providing any justification.

### Statistical Analysis

WPS 2019 software was used for data entry in this study. After the data were checked, they were entered into SPSS 19.0 for statistical analysis. Quantitative variables were described by the mean and standard deviation, while qualitative variables were described by the frequency distribution. T tests and variance homogeneity tests were used to compare the mental health status scores by participants’ demographic characteristics. The distribution of different mental statuses by demographic characteristics was determined by chi-square tests. The results were corrected for multiple comparisons. *P* < 0.05 was statistically significant.

## Result

### Demographic Characteristics

A total of 2,031 valid questionnaires from 29 provinces or political areas across China (except Hong Kong and Taiwan) were included in this research. Among the respondents, 85.52% were female and 79.17% were aged 20‑40 years. A total of 42.25% of the respondents were doctors, and 57.75% were nurses; 40.42% had experience combating similar outbreaks. A total of 36.53% had been exposed to patients with confirmed or suspected cases of COVID-19 (**Table 1**). One respondent was confirmed to be infected with COVID-19, two respondents had relatives who were suspected or confirmed to be infected with COVID-19, and five respondents were under isolation observation because of suspected exposure. The regional distribution of the survey population is shown in **Table 2**.

**Table 1:** Mental health status scores by demographic characteristics (mean ± SD) (DASS-21).

Characteristic variables	N(%)	Depression	Anxiety	Stress
**National average**	2031(100%)	4.36 ± 5.21	3.55 ± 4.66	7.03 ± 6.27
**Gender**				
Male	294(14.48%)	4.78 ± 5.73	3.17 ± 4.48	7.68 ± 6.10
Female	1737(85.52%)	4.29 ± 5.11	3.61 ± 4.68	6.93 ± 6.29
t		1.38	-1.51	1.90
P-value		0.16	0.13	0.05
**Age distribution**				
>60 years old	6 (0.30%)			
20-30	941 (46.33%)	4.17 ± 5.16	3.52 ± 4.67	6.34 ± 6.12
31-40	667 (32.84%)	4.30 ± 4.80	3.39 ± 4.33	7.33 ± 6.13
41-50	297 (14.62%)	5.15 ± 6.01	4.11 ± 5.13	8.24 ± 6.39
51-60	120 (5.91%)	4.50 ± 5.49	3.30 ± 5.00	7.88 ± 7.19
f		0.99	1.57	2.21
P-value		0.45	0.06	0.00
**Status**				
Doctor	858(42.25%)	4.76 ± 5.44	3.54 ± 4.62	7.64 ± 6.53
Nurse	1173(57.75%)	4.08 ± 5.02	3.56 ± 4.68	6.60 ± 6.03
t		2.85	-0.11	3.65
P-value		0.00	0.91	0.00
**Education**				
Below undergraduate	409(20.14%)	4.24 ± 5.64	3.59 ± 5.31	6.59 ± 6.54
Undergraduate	1345(66.22%)	4.34 ± 4.99	3.57 ± 4.55	7.14 ± 6.09
Postgraduate	277(13.64%)	4.69 ± 5.56	3.42 ± 4.08	7.18 ± 6.67
f		1.21	1.01	1.49
P-value		0.23	0.43	0.06
**Job title**				
Junior	1218 (59.97%)	4.20 ± 5.07	3.57 ± 4.66	6.57 ± 6.10
Intermediate	476 (23.44%)	4.41 ± 4.89	3.32 ± 4.22	7.29 ± 6.08
Senior	337 (16.59%)	4.92 ± 6.03	3.80 ± 5.17	8.36 ± 6.89
f		1.61	0.99	2.11
P-value		0.04	0.45	0.00
**Frontline**				
Yes	742(36.53%)	5.14 ± 5.89	4.19 ± 5.31	8.15 ± 6.82
No	1289(63.47%)	3.91 ± 4.65	3.17 ± 4.19	6.39 ± 5.84
t		4.80	4.46	5.89
P-value		0.00	0.00	0.00
**Experience with similar outbreaks**				
Yes	821(40.42%)	4.43 ± 5.19	3.54 ± 4.45	7.59 ± 6.31
No	1210(59.58%)	4.33 ± 5.23	3.56 ± 4.79	6.66 ± 6.21
t		0.42	-0.06	3.27
P-value		0.67	0.95	0.00
Regions^*^				
Chongqing	1603(78.93%)	4.17 ± 4.94	3.40 ± 4.33	6.69 ± 5.97
t		1.11	0.97	1.66
*P*-value		0.26	0.32	0.09
Sichuan	119(5.86%)	5.26 ± 5.58	4.23 ± 4.81	8.99 ± 6.76
t		-1.80	-1.55	-3.29
*P*-value		0.07	0.12	0.00
Shandong	117(5.76%)	5.21 ± 5.66	4.05 ± 5.03	8.13 ± 7.14
t		-1.69	-1.12	-1.63
*P*-value		0.08	0.26	0.10
Guizhou	42(2.07%)	4.95 ± 6.14	3.71 ± 5.42	7.76 ± 7.01
t		-0.71	-0.22	-0.74
*P*-value		0.47	0.82	0.45
Hubei	40(1.97%)	7.60 ± 8.63	6.65 ± 7.82	8.90 ± 8.97
t		-2.36	-2.49	-1.84
*P*-value		0.02	0.01	0.06
Beijing	32(1.58%)	3.93 ± 5.48	2.81 ± 3.96	6.31 ± 5.86
t		0.46	0.89	0.64
*P*-value		0.64	0.37	0.51

P < 0.05 was statistically significant.

*Compared the score of provinces to the national average respectively.

**Table 2:** Distribution of different mental statuses by demographic characteristics.

Characteristic variables	Depression(n%)					Anxiety(n%)					Stress(n%)				
	Normal	Mild	Moderate	Severe	Extremely severe	Normal	Mild	Moderate	Severe	Extremely severe	Normal	Mild	Moderate	Severe	Extremely severe
**Gender**															
Male	239 (81.29)	25 (8.50)	24 (8.16)	2 (0.68)	4 (1.36)	240 (81.63)	20 (6.80)	26 (8.84)	6 (2.04)	2 (0.68)	259 (88.09)	19 (6.46)	13 (4.42)	3 (1.02)	0 (0.00)
Female	1491 (85.83)	132 (7.59)	90 (5.18)	16 (0.92)	8 (0.46)	1419 (81.69)	104 (5.98)	165 (9.49)	28 (1.61)	21 (1.20)	1569 (90.32)	88 (5.06)	52 (2.99)	19 (1.09)	9 (0.51)
x^2^	8.47					1.28					4.20				
P-value	0.07					0.86					0.37				
**Age distribution**															
>60	6 (100)	0 (0.00)	0 (0.00)	0 (0.00)	0 (0.00)	6 (100)	0 (0.00)	0 (0.00)	0 (0.00)	0 (0.00)	6 (100)	0 (0.00)	0 (0.00)	0 (0.00)	0 (0.00)
20‑30	811 (86.18)	74 (7.86)	41 (4.36)	10 (1.06)	5 (0.53)	779 (82.78)	47 (4.99)	86 (9.14)	18 (1.91)	11 (1.17)	865 (91.92)	39 (4.14)	21 (2.23)	12 (1.28)	4 (0.43)
31‑40	572 (85.76)	51 (7.65)	38 (5.70)	4 (0.60)	2 (0.30)	547 (82.01)	46 (6.90)	58 (8.70)	10 (1.50)	6 (0.90)	597 (89.51)	39 (5.85)	24 (3.60)	5 (0.75)	2 (0.30)
41‑50	238 (80.13)	26 (8.75)	26 (8.75)	3 (1.01)	4 (1.35)	229 (77.10)	19 (6.40)	39 (13.13)	6 (2.02)	4 (1.35)	255 (85.86)	24 (8.08)	15 (5.05)	2 (0.67)	1 (0.34)
51‑60	103 (85.83)	6 (5.00)	9 (7.50)	1 (0.83)	1 (0.83)	98 (81.67)	12 (10.00)	8 (6.67)	0 (0.00)	2 (1.67)	105 (87.50)	5 (4.17)	5 (4.17)	3 (2.50)	2 (1.67)
x^2^	16.21					15.65					23.15				
P-value	0.18					0.20					0.02				
**Status**															
Doctor	712 (82.98)	76 (8.86)	55 (6.41)	8 (0.93)	7 (0.82)	693 (80.77)	68 (7.93)	77 (8.97)	10 (1.17)	10 (1.17)	756 (88.11)	49 (5.71)	40 (4.66)	8 (0.93)	5 (0.58)
Nurse	1018 (86.79)	81 (6.91)	59 (5.03)	10 (0.85)	5 (0.43)	966 (82.35)	56 (4.77)	114 (9.72)	24 (2.05)	13 (1.11)	1072 (91.39)	58 (4.94)	25 (2.13)	14 (1.19)	4 (0.34)
x^2^	6.27					10.81					12.02				
P-value	0.17					0.02					0.01				
**Education**															
Below undergraduate	356(87.04)	27(6.60)	16(3.91)	7(1.71)	3(0.73)	338(82.64)	18(4.40)	35(8.56)	9(2.20)	9(2.20)	374(91.44)	17(4.16)	7(1.71)	8(1.96)	3(0.73)
Undergraduate	1146 (85.20)	102 (7.58)	83(6.17)	7(0.52)	7(0.52)	1099(81.71)	82(6.10)	130 (9.67)	21(1.56)	13(0.97)	1205(89.59)	78(5.80)	44(3.27)	13(0.97)	5(0.37)
Postgraduate	228(82.31)	28(10.11)	15(5.42)	4(1.44)	2(0.72)	222(80.14)	24(8.66)	26(9.39)	4(1.44)	1(0.36)	249(89.89)	12(4.33)	14(5.05)	1(0.36)	1(0.36)
x^2^	15.526					12.222					13.443				
P-value	0.129					0.142					0.098				
Junior	1047 (85.96)	94 (7.72)	59 (4.84)	13 (1.07)	5 (0.41)	999 (82.02)	63 (5.17)	118 (9.69)	24 (1.97)	14 (1.15)	1114 (91.46)	52 (4.27)	33 (2.71)	15 (1.23)	4 (0.33)
Intermediate	403 (84.66)	42 (8.82)	26 (5.46)	4 (0.84)	1 (0.21)	393 (82.56)	35 (7.35)	38 (7.98)	6 (1.26)	4 (0.84)	424 (89.08)	35 (7.35)	13 (2.73)	3 (0.63)	1 (0.21)
Senior	280 (83.09)	21 (6.23)	29 (8.61)	1 (0.30)	6 (1.78)	267 (79.23)	26 (7.72)	35 (10.39)	4 (1.19)	5 (1.48)	290 (86.05)	20 (5.93)	19 (5.64)	4 (1.19)	4 (1.19)
x^2^	20.36					8.49					21.27				
P-value	0.00					0.38					0.00				
**Frontline**															
Yes	595 (80.19)	72 (9.70)	58 (7.82)	9 (1.21)	8 (1.08)	568 (76.55)	55 (7.41)	89 (11.99)	19 (2.56)	11 (1.48)	644 (86.79)	48 (6.47)	31 (4.18)	12 (1.62)	7 (0.94)
No	1135 (88.05)	85 (6.59)	56 (4.34)	9 (0.70)	4 (0.31)	1091 (84.64)	69 (5.35)	102 (7.91)	15 (1.16)	12 (0.93)	1184 (91.85)	59 (4.58)	34 (2.64)	10 (0.78)	2 (0.16)
x^2^	25.53					22.14					17.71				
P-value	0.00					0.00					0.00				
**Experience with similar outbreaks**															
Yes	704 (85.75)	54 (6.58)	51 (6.21)	8 (0.97)	4 (0.49)	668 (81.36)	47 (5.72)	89 (10.84)	10 (1.22)	7 (0.85)	723 (88.06)	60 (7.31)	28 (3.41)	6 (0.73)	4 (0.49)
No	1026 (84.79)	103 (8.51)	63 (5.21)	10 (0.83)	8 (0.66)	991 (81.90)	77 (6.36)	102 (8.43)	24 (1.98)	16 (1.32)	1105 (91.32)	47 (3.88)	37 (3.06)	16 (1.32)	5 (0.41)
x^2^	3.67					6.03					13.29				
P-value	0.45					0.19					0.01				

P < 0.05 was statistically significant.

### Descriptive Statistics of Anxiety, Depression, and Stress

As shown in **Table 1**, the total possible questionnaire score ranges from 0–42. The higher the score is, the more serious the degree of depression, anxiety, or stress. The results indicated that the mental states of most participants were normal. The average depression score in the whole cohort was 4.36 ± 5.21, the average anxiety score was 3.54 ± 4.66, and the average stress score was 7.03 ± 6.27 (**Table 1**). However, there were some differences among participants in terms of the degree of mental health problems. A total of 14.81%, 18.3%, and 9.98% of the whole cohort had symptoms of depression, anxiety and stress, respectively, and the rates of depression, anxiety and stress varied by demographic characteristics (**Table 2**).

### Comparison of Mental States by Demographic Characteristics

We found that men’s average stress scores were higher than women’s (*P*=0.05, **Table 1**). There was no significant difference in the average score of depression, anxiety or stress among medical staff with different educational levels, but trends towards an increased score of depression and stress were observed with increasing educational levels (**Table 1**). Compared with nurses, doctors had higher average scores of perceived stress (*P*=0.00) and depression (*P*=0.00) (**Table 1**), as well as a higher prevalence of anxiety (*P*=0.02) and stress (*P*=0.01) (**Table 2**). Compared with junior and intermediate workers, workers with senior job titles had higher average scores and probabilities of perceived stress (*P*=0.00 in **Tables 1** and **2**) and depression (*P*=0.04 in **Table 1**; *P*=0.00 in **Table 2**) (Multiple comparisons were conducted). Respondents between the ages of 31 and 60 years were more likely to report stress than those < 30 years old (*P*=0.00 in **Tables 1** and **2**) (Multiple comparisons were conducted); respondents over 60 years old showed normal levels for these three mental states (**Tables 1** and **2**). Respondents who had contact with patients with confirmed or suspected cases of COVID-19 working on the clinical frontlines were more likely to have higher perceived stress, anxiety, and depression (*P*=0.00 in **Tables 1** and **2**). In addition, respondents who had previous experience combating similar outbreaks showed higher perceived stress (*P*=0.00 in **Table 1**; *P*=0.01 in **Table 2**). There was no significant difference between the scores of respondents from four provinces (Chongqing, Shandong, Guizhou, and Beijing) and the national average, while respondents from other provinces, especially Hubei Province, had higher scores than the national average (**Table 1**).

## Discussion

With the outbreak and spread of the novel coronavirus epidemic in 2019, the number of confirmed cases is rising rapidly daily. To quickly control the epidemic and save the lives of infected patients, Chinese medical workers have been extremely busy doing hard work in the past 5 months and have made great sacrifices. As of Feb. 24, according to the report of the joint inspection expert group of China and the WHO, 3,387 medical staff from 476 medical institutions in China have been infected with COVID-19 (2,055 confirmed cases, 1,070 clinically diagnosed cases, and 157 suspected cases); more than 90% of infected medical staff (3,062 cases) came from Hubei Province, and 24 of them have died. As was previously known, paediatric medical staff in China face several hurdles, including high occupational risk, multiple conflicts, heavy workload and long working hours. Since the outbreak, due to overwork and the potential risk of infection, paediatric medical staff may be under great psychological pressure that may not disappear immediately with the end of the epidemic. Some of them are at risk of experiencing posttraumatic stress disorder symptoms.

To understand psychological problems among paediatric medical workers during this epidemic and to explore effective psychological intervention measures, we surveyed 2,031 paediatric medical workers from 29 provinces or political areas in China by using an Internet application. We found that 14.81% of the respondents had depressive symptoms, 18.3% had anxiety symptoms, and 9.98% had stress symptoms. The rate of depressive symptoms is higher than that found in a previous study on the general Chinese population prevalence of depressive symptoms (13.5%) (8).

A comparison of the DASS-21 scores of paediatric medical staff in different regions revealed that among the provinces with a sample size of more than 20 respondents, there was no significant difference between the scores of respondents from four provinces (Chongqing, Shangdong, Guizhou and Beijing, respectively) and the national average. Among these provinces, respondents from Chongqing and Beijing scored slightly lower than the national average, while those from Shangdong and Guizhouo scored slightly higher than the national average. In particular, the scores of respondents from Hubei were significantly higher than the national level, likely due to the serious situation in Hubei (9). Chongqing and Beijing are both municipalities that are directly under the central government of China. The numbers of confirmed cases in these two cities were among the highest in the country, but the perceived stress of paediatric doctors and nurses was generally low, which may be related to the professional medical backgrounds of medical staff and their more objective cognition of disease risk (10). This finding may also be attributed to the early awareness of the particularity and severity of the epidemic by local governments as well as the timely and detailed working guidelines they developed.

Great differences in the occurrence of psychological problems among different medical workers have been documented in previous studies (11–13). Our study also found that frontline paediatric medical workers who were fighting against the epidemic had higher stress, anxiety, and depression because they were the highest-risk group for COVID-19 infection, and medical staff who had experienced similar events showed higher levels of perceived stress due to their higher awareness of the risk of disease. In addition, men’s perceived stress perception was higher than women’s, and doctors had higher perceived stress and depression levels than nurses. By comparing the emotional states of paediatric medical staff in different age groups, we found that stress were most likely to occur among 31- to 60-year-olds. We also found that the more senior the job title was, the higher the depression and stress scores. An analysis of the reasons for these findings showed that most of the paediatric medical staff between 31–60 years old, with senior job titles, especially in men and doctors, were the main persons in charge of clinical work, and they needed to take more responsibility and bear greater pressure. These meaningful social and demographic characteristics analysed above can guide us to develop psychological intervention programmes that are tailored to address the different emotional symptoms and needs of paediatric medical workers.

### Limitations of This Study

Our study had several limitations. First, although a national sample was obtained, the sample of respondents affected by COVID-19 as of the time of the survey was not large enough. Second, we used online questionnaires through social network software and could not fully explain the questionnaire to the respondents. Third, we did not have mental health data from non-epidemic periods for comparison; conducting such a comparison will be our next step. These factors may have biased the results of the study.

## Conclusion

During the COVID-19 outbreak, medical staff have taken on high-intensity work every day. In addition to suffering physical exhaustion, they have suffered psychologically. Similar to everyone else, they feel depressed, anxious, and stressed. Paediatric medical staff are a group that experiences high psychological pressure on Chinese medical teams, which may have further increased their psychological burden since the outbreak. Depression, anxiety, and stress symptoms due to natural disasters can be prevented from becoming chronic problems through appropriate mental health management, prompt responses, efforts to meet individualized needs, and efforts to provide appropriate interventions after the identification of symptoms of anxiety, depression, or stress (14). Understanding the emotional problems that different groups may face and implementing corresponding psychological intervention programmes to protect the psychological health of paediatric anti-epidemic personnel can have a profound impact on the fight against the epidemic and workers’ own long-term health (9).

## Data Availability Statement

The original contributions presented in the study are included in the article/supplementary material, further inquiries can be directed to the corresponding author.

## Ethics Statement

The studies involving human participants were reviewed and approved by Ethics Committee of the Children’s Hospital of Chongqing Medical University. Written informed consent for participation was not required for this study in accordance with the national legislation and the institutional requirements.

## Author Contributions

LB and YS conceptualized and designed the study, drafted the manuscript, and approved the final manuscript as submitted. LW and YL designed the data collection instruments, coordinated and supervised the data collection, carried out the initial analyses, drafted the manuscript, and approved the final manuscript as submitted. LC and XZ coordinated and supervised the data collection and approved the final manuscript as submitted.

## Conflict of Interest

The authors declare that the research was conducted in the absence of any commercial or financial relationships that could be construed as a potential conflict of interest.
